# Deciphering the interplay between SETD2 mediated H3K36me3 and RNA N6-methyladenosine in clear cell renal cell carcinoma (ccRCC)

**DOI:** 10.1080/15592294.2025.2456418

**Published:** 2025-01-28

**Authors:** Shafiq Shaikh, Xia Zhao, Ryan T. Wagner, Xiaoyu Pan, Ryan A. Hlady, Liguo Wang, Thai H. Ho, Keith D. Robertson

**Affiliations:** a Department of Biochemistry and Molecular Biology, Mayo Clinic, Rochester, MN, USA; b Department of Molecular Pharmacology and Experimental Therapeutics, Mayo Clinic, Rochester, MN, USA; c Division of Computational Biology, Mayo Clinic College of Medicine and Science, Rochester, MN, USA; d Division of Hematology and Oncology, Medical University of South Carolina, Charleston, SC, USA

**Keywords:** H3K36me3, SETD2, N6-methyladenosine (m6A), METTL3, STM2457, H3K27ac, renal cell cancer, ccRCC

## Abstract

RNA N6-methyladenosine (m6A) plays diverse roles in RNA metabolism and its deregulation contributes to tumor initiation and progression. Clear cell renal cell carcinoma (ccRCC) is characterized by near ubiquitous loss of *VHL* followed by mutations in epigenetic regulators *PBRM1*, *SETD2*, and *BAP1*. Mutations in *SETD2*, a histone H3 lysine 36 trimethylase (H3K36me3), are associated with reduced survival, greater metastatic propensity, and metabolic reprogramming. While m6A and H3K36me3 deregulation are separately implicated in renal tumorigenesis, H3K36me3 may participate directly in m6A targeting, but the m6A-H3K36me3 interplay has not been investigated in the context of ccRCC. Using RCC-relevant SETD2 isogenic knockout and rescue cell line models, we demonstrate a dynamic redistribution of m6A in the SETD2 depleted transcriptome, with a subset of transcripts involved in metabolic reprogramming demonstrating SETD2 dependent m6A and expression level changes. Using a panel of six histone modifications we show that m6A redistributes to regions enriched in gained active enhancers upon *SETD2* inactivation. Finally, we demonstrate a reversal of transcriptomic programs involved in SETD2 loss mediated metabolic reprogramming, and reduced cell viability through pharmacologic inhibition or genetic ablation of m6A writer METTL3 specific to SETD2 deficient cells. Thus, targeting m6A may represent a novel therapeutic vulnerability in *SETD2* mutant ccRCC.

## Introduction

Renal cell carcinoma (RCC) is the 8^th^ leading cause of cancer death in the US and among the 10 most frequently detected cancers worldwide [1,2]. Clear cell renal cell carcinoma (ccRCC) is the most common subtype of RCC accounting for 80% of cases [3]. CcRCC is characterized by near universal loss of chromosome 3p and the *VHL* gene, an E3 ubiquitin ligase that ubiquitylates and targets hypoxia inducible factors HIF1α and HIF2α for proteasome-mediated degradation under normoxic conditions [4]. While *VHL* loss is the key initiating event, ccRCC progression is driven by subsequent loss of epigenetic regulators *SETD2*, *PBRM1*, and *BAP1*, which result in a profoundly disrupted epigenome leading to aberrant gene expression and deregulated cell growth control [5–7].

*SETD2* is a histone H3 lysine 36 trimethylase (H3K36me3); its loss in ccRCC leads to global H3K36me3 depletion accompanied by marked re-organization of the epigenome, particularly the repressive H3K27me3 mark deposited by polycomb repressive complex 2 (PRC2) and increased genomic accessibility accompanied by ectopic enhancer activation [8,9]. SETD2 loss has also been linked to defective DNA repair, altered mRNA splicing, and metabolic reprogramming [3,10,11]. *SETD2* mutations occur in 15–20% of primary ccRCC but increase to nearly 60% in ccRCC metastases [6,9,12–14]. Loss of H3K36me3 due to *SETD2* mutation is associated with increased brain, lung, and bone metastasis, and reduced overall and progression free survival in ccRCC patients [11,13,15]. While mutations in *BAP1* individually or in combination with *PBRM1* are associated with worse cancer specific survival, *SETD2* mutations are associated with increased disease recurrence post resection [2]. These findings collectively implicate *SETD2* mutations in promoting metastasis and engendering drug resistance during ccRCC progression, emphasizing the need to better understand molecular pathways altered in SETD2 deficient ccRCC [2].

Methylation of the N6 position of adenine (m6A) is the most abundant post-transcriptional modification of mRNA and modulates a wide array of cellular processes such as cell death, RNA splicing, transcriptional regulation, mRNA stability, translation, and DNA repair [16–21]. Many of the effects of m6A are mediated by m6A readers including YTHDC1, YTHDF1–3, and IGF2BP1–3, which recognize and process the m6A modified transcript for degradation or promote its translation [19,21]. The m6A core methyltransferase complex consisting of the heterodimeric METTL3 – METTL14 complex, which guides m6A modifications onto nascent mRNA transcripts within the consensus RRACH motif, has been shown to be recruited to H3K36me3 marked chromatin regions [22]. Other proteins like WTAP, VIRMA, and RBM15/15B function to recruit the METTL3/METTL14 complex to nuclear speckles or to specific transcripts for methylation [22,23]. Accumulating evidence implicates deregulated m6A patterns in promoting cancer cell proliferation, migration and invasion, as well as inducing metabolic deregulation [24–26]. Oncogenic functions of m6A are initiated by increased METTL3 expression, which leads to increased m6A in oncogenic targets followed by recruitment of m6A readers such as YTHDF1 to enhance transcript stability and translational efficiency of m6A modified transcripts [24–26]. Consistent with this, METTL3 levels are elevated in ccRCC relative to adjacent normal tissue and have been linked to several key aspects of ccRCC biology including fatty acid biosynthesis, PI3K/AKT signaling, and regulation of the cell cycle kinase CDK4 [27–29].

Given that a deleterious role for m6A/METTL3 deregulation in ccRCC biology has been established, and that m6A may be targeted in part by SETD2 mediated H3K36me3, which is frequently lost in ccRCC and linked to poor outcome, we sought to investigate the interplay between SETD2 and m6A patterning/localization in the transcriptome of *SETD2* deficient ccRCC. Using RCC relevant isogenic models, we show that SETD2 loss leads to global m6A redistribution manifested by both m6A gains and losses. Additionally, we identify a core group of genes that are concomitantly m6A hypermethylated and transcriptionally upregulated upon *SETD2* inactivation, which are enriched in pathways known to promote metabolic reprogramming in SETD2 deficient ccRCC [30]. Furthermore, we track differential m6A hypermethylation to genomic regions enriched in *SETD2* mutation-associated acquired active enhancer marks H3K27ac and H3K4me1. To validate the functional contribution of m6A hypermethylated transcripts to cell survival and proliferation in ccRCC, we generated isogenic *METTL3* knockout (KO) cells in both a *SETD2* WT and SETD2 KO ccRCC cell line context and demonstrate that METTL3 inactivation, or treatment with the METTL3 inhibitor STM2457, preferentially reduced cell growth and colony forming potential, increased apoptosis, and downregulated key genes in pathways that promote metabolic reprogramming in a *SETD2* mutant ccRCC cell line. As such, targeting METTL3 may represent a novel therapeutic vulnerability for *SETD2* mutant ccRCC.

## Materials and methods

### Cell culture, drug treatments, and cell growth assays

The 786-O ccRCC cell line (referred to as the wild-type (WT) or parental line), hTERT immortalized renal proximal tubule epithelial cells (RPTEC, referred to as the wild-type (WT) parental line), and HEK293T were cultured as described [31]. The 786-O *SETD2* KO isogenic clones (referred to here as SETD2KO–1 and SETD2KO–2) have been described [31,32]. The parent (WT), isogenic RPTEC SETD2KO–1 and SETD2KO–2, and full-length (FL) FLAG-SETD2 rescue RPTEC (RPTEC SETD2KO-1 R) lines have also been described [31]. Rescue of 786-O SETD2KO–1 was performed similarly using a tagged SETD2 construct and is referred to here as 786-O SETD2KO-1 R. 786-O WT and SETD2KO–2 cells were transduced with an H2A – EGFP fusion gene expressing lentivirus before treatment with 20 µM METTL3 inhibitor STM2457. The cell proliferation assay was performed by plating cells into a 48-well plate at 1000 cells/well and replacing media containing the above-mentioned concentration of STM2457 every day for cell proliferation and colony formation assays. Cells were similarly treated with STM2457 for 3 days before quantifying caspase 3/7 activation as described below. The number of cells with EGFP positive nuclei was quantified to assess cell proliferation using an Incucyte S3 high-throughput live-cell microscope (Sartorius). Cell proliferation and colony formation assays for 786-O *METTL3* KO (referred to as 786-O METTL3KO) and 786-O *SETD2*/*METTL3* double KO (referred to as 786-O DKO) as well as guide RNA control transduced 786-O WT (referred to as 786-O WT Ctrl) and 786-O SETD2KO–2 (referred to as 786-O SETD2KO–2 Ctrl) cells were performed by seeding cells onto 48- and 6-well plates at densities of 1000 and 200 cells/well respectively. Colonies were allowed to grow for approximately 10 days before being fixed, stained with 0.5% crystal violet and quantified. Cell proliferation in 786-O WT *METTL3* KO and 786-O DKO as well as their respective controls 786-O WT Ctrl and 786-O SETD2KO–2 Ctrl cells was assessed using the Incucyte S3 by quantifying the phase area occupied by cells with the adherent cell-by-cell analysis program. *METTL3* KO and control 786-O cells were plated onto 6-well plates and allowed to equilibrate for at least 1 day before being processed for caspase 3/7 quantification as described below.

### Gene targeting and generation of isogenic CRISPR KO clones

CRISPR/CAS9 inactivation of *SETD2* in RPTEC and 786-O cells is described elsewhere [31]. *METTL3* inactivation in 786-O WT and SETD2KO–2 cells was performed by directing CAS9 activity to *METTL3* exon 3 (sgRNA: GAGCTTGGAATGGTCAGCAT) to generate biallelic frameshift mutations ablating gene function. The sgRNAs were annealed and ligated using complementary overhangs to an in-house generated CRISPR/CAS9 targeting vector (Supplemental Figure S8) made with the pLVX lentiviral backbone and the guide RNA expressed under control of the mouse U6 promoter. *METTL3* was targeted for inactivation by transducing ~ 5 X 10^4^ cells with METTL3 guide RNA expressing lentivirus. Individual clones were subsequently expanded and screened for INDELs by PCR amplification and Sanger sequencing spanning the exon 3 target sequence. Three independent METTL3 KO clones were derived and validated in WT 786-O cells (clones A1, A2, and A3, referred to here as KO1, KO2, and KO3, respectively) and two independent METTL3 KO clones were derived in 786-O SETD2KO–2 cells (clones C1 and C2, referred to here as DKO-1 and DKO-2). Mutational profiles of each clone were determined by deconvolution of the sequencing traces using Synthego’s ICE analysis tool. Individual clones containing frameshift mutations were functionally validated for *SETD2* or *METTL3* ablation by Western blot analysis and global H3K36me3 and m6A levels, respectively.

### Lentivirus preparation and transduction

Packaging of lentiviral particles was carried out by lipid-based transient transfection 24 hrs following plating of 293T cells (plating density ~ 1 × 10^5^ cells/cm^2^). Lentiviral transfer vectors were mixed with 2nd generation packaging plasmids psPAX2 (Addgene #12260) and pMD2.g (Addgene #12259) at a molar ratio of 3:2:1 and delivered to 293T cells using JetOptimus transfection reagent (Polyplus # 101000051) with a DNA:lipid ratio of 1:2. Lentiviral supernatant was collected 72 hrs post transfection and filtered using a 0.45 µm polyether sulfone syringe filter (Fisher Scientific 13-100-107). Lentivirus was concentrated when required by ultracentrifugation at 25,000 RPM for 2 hrs at 4°C and resuspended in 0.5 ml DMEM. Lentiviral transduction of target cells was performed at an MOI of 1–5 depending on the application for 48 hrs before removal of the lentivirus and initiation of drug selection.

### RNA extraction and qRT-PCR

Trizol was used for RNA extractions as previously described [33]. Total RNA was purified from DNA contaminants using the Qiagen RNase-free DNase SET (Qiagen #79254) with the recommended protocol followed by ethanol precipitation to extract purified RNA. Purified RNA (1 µg) was used to synthesize cDNA using the high-capacity cDNA reverse transcription kit according to the manufacturer’s protocol (Applied Biosystems #4368813). Gene specific transcription for METTL3 was quantified on a CFX96 qPCR machine (BioRad) using METTL3 specific primers (Supplemental Table S1) and SYBRgreen – qPCR quantification. Gene amplification values (Ct) were normalized using delta-delta Ct methodology.

### Methylated RNA immunoprecipitation sequencing (MeRIP-seq) and MeRIP-QPCR

MeRIP-seq was performed as described [34] on the samples listed in Supplemental Table S3. Briefly, 23 ng of *E. coli* K-12 RNA was added to every 5 µg of total cellular RNA as a spike-in for downstream normalization. Spike-in and total RNA (50 µg), were fragmented to 130 – 250nt size using the NEBnext® RNA fragmentation module according to the manufacturer protocol and then subjected to ethanol precipitation. After sequestering 2 µg of RNA for further processing as input, the remaining RNA was resuspended in 1X IP buffer (150 mm NaCl, 10 mm Tris-HCl [pH 7.5], 0.1% IGEPAL CA-630, nuclease free H_2_O) with RNase inhibitor. Protein A magnetic beads (NEB #S1425S) were pre-blocked by incubating with total RNA for 1 hour at 4°C. Protein A magnetic beads (30 µl) were then washed and primed with 5 µg of anti-m6A antibody (Millipore #ABE572) by incubating the beads with the antibody for 6 hrs at 4°C. Total RNA with the spike in mix was incubated with m6A antibody primed protein A beads overnight at 4°C. Beads were then washed once in 1X IP buffer, low salt IP buffer (50 mm NaCl, 10 mm Tris-HCl [pH 7.5], 0.1% IGEPAL CA-630 in nuclease free H_2_O), and high salt IP buffer (500 mm NaCl, 10 mm Tris-HCl [pH 7.5], 0.1% IGEPAL CA-630 in nuclease-free H_2_O) before resuspending beads in Trizol. RNA bound to the beads was extracted using Trizol extraction. IP as well as input total RNA (90 ng) was used to synthesize cDNA using Promega GoScript reverse transcription system (Promega, #A5001) as described previously [35], with 5 µl of cDNA per replicate being used to perform QPCR for target genes using peak specific primers (Supplemental Table S2). The m6A peak (IP) amplification values were normalized to background expression (Input) using delta-delta Ct methodology.

### Mass spectrometry

MRNA was isolated from total RNA using the NEBnext poly(A) mRNA magnetic isolation module (NEB #E7490) and quantified using the Qubit RNA HS Assay kit (ThermoFisher #Q32855). Purified mRNA was then hydrolyzed to single nucleosides, which were dephosphorylated and deproteinized using the Sartorius 10,000-Da MWCO spin filter. Nucleoside mixtures were analyzed using an Agilent 6460 QQQ triple quadrupole mass spectrometer with an Agilent 1260 hPLC system in multi reaction monitoring (MRM) mode at Arraystar (Rockville, MD). LC-MS data were acquired and analyzed using Agilent qualitative analysis software. MRM peaks of m6A modified nucleoside were normalized to total adenosine residues quantified.

### MeRIP-seq, ChIP-seq, and RNA-seq library prep

RNA-seq libraries for all samples (Supplemental Table S3) were prepared using the TruSeq Stranded mRNA library kit using the manufacturer’s instructions at the University of Minnesota Genomics Center (Minneapolis, MN). ChIP-seq libraries (Supplemental Table S3) were prepared as described previously [31] using antibodies listed in Supplemental Table S4. MeRIP-seq libraries for the indicated samples in Supplemental Table S3 were prepared using the SMARTer stranded total RNA-seq kit V3 – pico mammalian input using the manufacturer’s instruction at the University of Minnesota Genomics Center.

### MeRIP-seq, ChIP-seq, and RNA-seq bioinformatic analysis

ChIP-seq and RNA-seq data were analyzed as described [31]. MeRIP-seq and RNA-seq libraries were sequenced on a NovaSeq 6000, 150 bp paired end run to at least 20 million reads per sample at the University of Minnesota Genomics Center. *Drosophila* S2 chromatin was spiked into all chromatin preparations before immunoprecipitation for downstream normalization. ChIP-seq libraries were sequenced at the Mayo Clinic Medical Genome Facility on an Illumina HiSeq 4000. Processing and analysis of ChIP-seq and RNA-seq data sets has been described elsewhere [36]. Briefly, ChIP-seq reads were mapped and aligned using the spiker custom analysis tool (https://github.com/liguowang/spiker), which uses a combined custom human and *Drosophila* index to parse out human and *Drosophila* reads using the bowtie2 algorithm. Scaling factors were generated by quantifying *Drosophila*-human read ratios and applied to each sample for normalization. DESeq2 was used to call significantly differential peaks with log2FoldChange ≥ or ≤ +1 or −1, respectively, and an adjusted p-value of < or = 0.05 called significantly different. For MeRIP-seq and RNA-seq datasets, reads were mapped and aligned using the STAR aligner followed by processing with HTSeq 2.0.3 for quantification; reads were normalized using the scaling factors generated from *E. coli K12* RNA spike-in. M6A peaks were identified for MeRIP-seq using MACS2 2.2.9.1 as in [34] by calling peaks statistically significant (qvalue < or = 0.05) from the background RNA expression (Input). Differential peaks for MeRIP-seq and differential RNA expression analysis were performed using DESeq2 1.20.1. ChromHMM v1.25 was used to generate chromatin state annotations as described previously [37]. Enrichment of overlapping genomic intervals was tested using the Genomic Association test (GAT) [38].

### Western blotting

Whole cell lysates were generated from target cell lines by resuspending pelleted cells in 1X RIPA lysis buffer (Abcam #ab156034) supplemented with protease inhibitor cocktail (Roche #11836170001). Protein concentrations were measured using the BCA protein assay (Thermo Scientific #23227); 30 µg of protein was loaded into each well of 4–20% precast gradient polyacrylamide gels (BioRad #461091) followed by wet-transfer onto PVDF membrane (25 mm Tris-HCl pH 7.5, 192 mm glycine, 10% methanol, 0.01% SDS) at 20 V overnight at 4°C. Membranes were blotted with α-METTL3 antibody (Proteintech 15,073-I-AP) at 1:1000 concentration overnight at 4°C. Validation of SETD2KO and rescue lines using histone extracts was performed as described previously [31], using antibodies described in Supplemental Table S4. Western blots were imaged using the Odyssey Xf imaging system (LI-COR) after secondary antibody incubation at room temperature for 1 hr.

### Apoptosis and colony formation assays

After cells were pelleted and washed once with PBS, they were assessed for caspase 3/7 activation by staining with Cellevent Caspase-3/7 detection reagent (FITC #C10423 or Texas red #C10430) according to the manufacturer’s instructions. Fluorescent cells with activated caspase 3/7 were detected using a LSR Fortessa X-20 flow cytometer (BD Biosciences). The percentage of cells positive for caspase 3/7 cleaved peptide were determined by applying appropriate fluorophore specific gating criteria using FlowJo analysis software (FlowJo, LLC). The percentage of caspase 3/7 positive METTL3KO cells were normalized to the percentage caspase 3/7 positive respective control cell lines. 786-O WT and *SETD2* KO cells either treated with METTL3 inhibitor STM2457 or genetically depleted for METTL3 (*METTL3* KO) were seeded at a clonal density of 20 cells/cm^2^. Cells were allowed to grow for 7–10 days before being fixed with ice-cold methanol (10 minutes at −20°C) and stained with crystal violet (0.5% crystal violet, 25% methanol; 10 minutes room temp). Stained cells were washed with water to remove excess crystal violet and the number of colonies counted.

### Statistical analysis

All quantifications were analyzed for statistical significance using one-way anova with *p* values corrected for multiple comparisons using Sidaks multiple comparison test where appropriate. All data is presented as mean ±SEM with p ≤ 0.05 considered statistically significant. The statistical significance is denoted as *p < 0.05, **p < 0.01, ***p < 0.001 and ****p < 0.0001. Relevant pathways enriched in our target gene lists were analyzed using EnrichR [39]. Briefly, mean rank and standard deviation from expected rank in the gene set library was obtained by performing Fisher exact test on random input gene lists. Then Z scores calculated from the deviation from expected rank were combined with the *p* value from Fisher exact test to obtain a combined score, which was used to determine enrichment. The number of samples or replicates used in the analyses is mentioned in the figure legends. Log rank test was performed on median separated high and low gene expression groups for TCGA-KIRC tumor samples to determine statistical significance between high- and low-expression tumor samples for the genes mentioned. Correlation coefficient R and corresponding *p* values represents Pearson’s correlation test. Homer was used to calculate enrichment of consensus motifs in top the 3000 786-O WT called m6A peaks in comparison to randomly generated background sequences with *p* value representing default statistical analysis tools used by HOMER. Log2FoldChange gene expression and m6A changes analyzed for comparisons between 786-O and RPTEC *SETD2* KO and WT cells are referred to as SETD2 KO vs WT, and comparisons between 786-O and RPTEC *SETD2* rescue and *SETD2* KO cells are referred to as SETD2 resc vs KO.

## Results

### SETD2 loss causes global redistribution of m6A within the epitranscriptome

**Figure 1.**
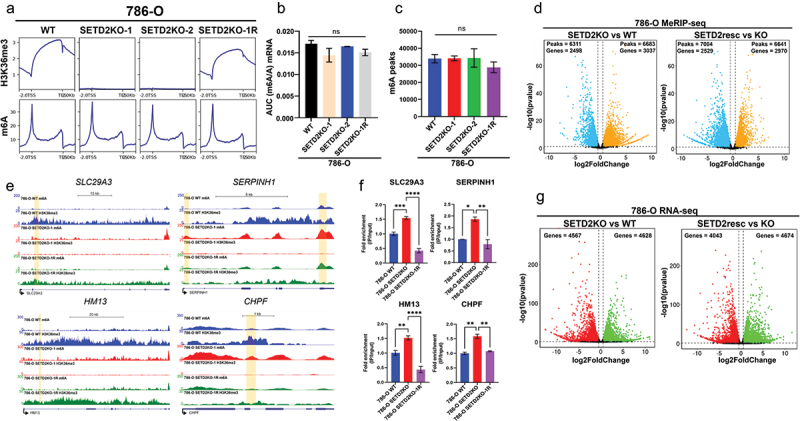
Changes in m6A distribution across the epitranscriptome upon *SETD2* inactivation and H3K36me3 depletion. (a) tag density plot showing average m6A and H3K36me3 peak distributions across gene bodies ± 2kb. (b) LC-MS area under the curve (AUC) quantification (m6A/A) for 786-O WT, *SETD2*KO-1 and *SETD2*KO-2, and *SETD2*KO-1 R (rescue) mRNA (*n* = 2). (c) total number of m6A peaks called for the indicated cell lines after MeRIP-seq (*n* = 2). (d) volcano plots of differential m6A peaks in 786-O *SETD2* KO vs WT and *SETD2* KO rescue (resc) vs KO comparisons. M6A hypermethylated (Log2FoldChange ≥0.5, *p*-value ≤0.05, orange), m6A hypomethylated (Log2FoldChange ≤0.5, *p*-value ≤0.05, blue), and unchanged (black). (e) gene level browser view of *SLC29A3, SERPINH1, HM13 and CHPF* loci showing m6A and H3K36me3 levels in 786-O WT (blue), *SETD2* KO-1 (red), and *SETD2*KO-1 R (green) with tan colored boxes highlighting called peaks. (f) MeRIP-qPCR validation of *SLC29A3*, *SERPINH1*, *HM13* and *CHPF* showing relative fold enrichment of m6A in the IP fraction compared to the input for 786-O WT (blue), 786-O *SETD2*KO (red) and 786-O *SETD2*KO-1 R (magenta). (g) volcano plots depicting differentially expressed genes in 786-O *SETD2* KO vs WT and *SETD2* KO resc vs KO comparisons, upregulated (Log2FoldChange ≥0.5, *p*-value ≤0.05, green), downregulated (Log2FoldChange ≤0.5, *p*-value ≤0.05, red), and unchanged (black).

The m6A writer METTL3, which forms a crucial component of the core methyltransferase complex [22], is significantly overexpressed in ccRCC compared to normal kidney and higher expression correlates with reduced ccRCC survival [40,41], suggesting a role for m6A in ccRCC progression (Supplemental Figures S1A,B). To understand the interplay between SETD2 status and m6A localization in RCC relevant models, we made use of previously generated isogenic *SETD2* KOs in the ccRCC cell line 786-O (referred to as SETD2KO–1 and SETD2KO–2) and the surrogate normal immortalized renal proximal tubule cell line RPTEC (SETD2 KOs also referred to as SETD2KO–1 and SETD2KO–2). To better define direct versus indirect changes in m6A localization driven by SETD2 status, we rescued one of each of the *SETD2* KO clones (SETD2KO–1 for 786-O [42] and SETD2KO–1 for RPTEC [31]) with a full-length wild-type FLAG-tagged SETD2 transgene followed by clonal derivation. We reasoned that changes induced by SETD2 loss but rescued by SETD2 re-expression are likely the most direct targets of SETD2/H3K36me3 activity. Western blot as well as ChIP-seq analysis shows complete depletion of the H3K36me3 mark in *SETD2* KO 786-O clones as well as its restoration within gene bodies upon rescue with the FL-SETD2 transgene (Figure 1a, top, and Supplemental Figures S1CE). Similar depletion of the H3K36me3 mark in SETD2KO RPTEC clones and its rescue with FL-SETD2 transgene expression has been demonstrated previously [31]. To assess the impact of SETD2 depletion on global m6A levels, we performed mass spectrometry on purified mRNA using the 786-O isogenic series. This analysis revealed no significant differences in global m6A levels with *SETD2* KO or rescue (Figure 1b). To examine specific gene level changes in m6A patterns we performed methylated RNA immunoprecipitation-sequencing (MeRIP-seq) in the 786-O and RPTEC isogenic series. Tag density plots across all genes show that while H3K36me3 is depleted with *SETD2* KO and restored upon SETD2 rescue, m6A levels averaged across all genes remained largely unaffected by *SETD2* status in 786-O cell lines (Figure 1a, compare top and bottom panels) and the *SETD2* isogenic RPTEC series (Supplemental Figures S1C,D). Consistent with known literature, we observed significant enrichment of the m6A consensus motif ‘RRACH’ after performing HOMER motif analysis on top 3000 m6A peaks in each cell line model (Supplemental Figure S1F). Moreover, we did not observe a significant difference in the total number of m6A peaks among the 786-O and RPTEC series isogenic for SETD2 loss and rescue (Figure 1c, Supplemental Figure S1G), nor was there significant changes in METTL3 expression with *SETD2* KO in 786-O cells (Supplemental Figure S1H). RPTEC SETD2KO–1 showed a modest yet significant reduction in METTL3 expression compared to the parental cells (Supplemental Figure S1H). Thus, our results indicate that loss of SETD2 in RCC relevant models does not result in significant transcriptome-wide m6A depletion. This is further supported by the consistent levels of METTL3 expression in SETD2 WT compared to SETD2KO clones for 786-O and RPTEC cell lines, which further precludes differential METTL3 levels resulting from transcript level m6A changes with SETD2KO.

Given that global losses in m6A with SETD2 inactivation were not observed, as might have been expected based on a prior study using different cellular models [22], we investigated transcript level differences in m6A in *SETD2* isogenic lines. MeRIP-seq of *SETD2* WT and *SETD2* depleted/rescued cells revealed a marked redistribution of m6A peaks in both the 786-O and RPTEC models. For example, *SETD2* KO in 786-O cells resulted in 6683 gained m6A peaks (corresponding to 3037 genes) and loss of 6311 peaks (corresponding to 2498 genes), while rescue of SETD2 in 786-O *SETD2* KO cells resulted in gain of 6641 peaks (corresponding to 2970 genes) and loss of 7004 peaks (corresponding to 2529 genes) (Figure 1d). Similarly, in the RPTEC *SETD2* isogenic series, differential MeRIP-seq analysis revealed both widespread m6A hypermethylation (5106 peaks corresponding to 2068 genes) and m6A hypomethylation events upon SETD2 inactivation (3891 peaks/1973 genes) that were also largely rescued upon re-expression of SETD2 in the KO cells (Supplemental Figure S2A) consistent with results in the 786-O model. Representative genome browser views illustrate m6A hypermethylation events at the *SLC29A3*, *SERPINH1*, *HM13*, and *CHPF* loci in 786-O cell lines, and *POU2F2* loci in the RPTEC cell lines. The *MAL* locus, for comparison, illustrates a representative m6A hypomethylation event in SETD2KO RPTEC cell line (Figure 1e, Supplemental Figure S2B). Similarly, MeRIP-QPCR performed using m6A peak specific primers showed positive fold enrichment in 786-O SETD2KO IP fraction (input normalized) compared to 786-O parent IP fraction (input normalized) (Figure 1f), confirming the MeRIP-seq-based findings. Notably, we also observed increased gene expression for *SLC29A3*, *SERPINH1*, *HM13* and *CHPF* with *SETD2* KO that was reversed, along with m6A enrichment upon *SETD2* rescue (Figure 1f and Supplemental Figure S1I). The reversal of *SETD2* KO mediated m6A methylation and gene expression signatures in 786-O and RPTEC *SETD2* rescue lines (Figures 1e,f and Supplemental Figure S1I and S2B) suggests that *SETD2* activity tracks tightly with redistribution (both loss and gain) of transcript level m6A and gene expression patterns.

To assess the relationship between differential m6A patterns and differential gene expression, we performed RNA-seq on both *SETD2* isogenic models. In 786-O cells, SETD2 inactivation resulted in 4628 upregulated and 4567 downregulated genes that, again, were largely reversed upon SETD2 rescue (Figure 1g). Comparable results for gene expression changes were observed in the RPTEC isogenic series (Supplemental Figure S2A, bottom panel). Pearson’s correlation test between differential m6A and differential expression revealed a robust positive correlation in the *SETD2* KO vs WT comparison for 786-O (*R* = 0.47, *p* < 0.001), the similar comparison for RPTEC (*R* = 0.58, *p* < 0.001), as well as for the *SETD2* KO resc vs KO comparison for 786-O (*R* = 0.71, *p* < 0.001) and RPTEC (*R* = 0.48, *p* < 0.001, Supplemental Figure S2C). This finding suggests that RNA expression changes track positively with m6A changes. While increases in m6A could be regarded as merely a byproduct of increased transcription, two lines of evidence indicate this is not the case. Firstly, all m6A called peaks are adjusted for input or background RNA expression. Secondly, out of a total of 2660 genes upregulated in the *SETD2* KO vs WT comparison in 786-O cell lines only 1099 genes (~50%) are m6A hypermethylated with SETD2 inactivation. Similarly, in the SETD2 resc vs KO comparison in 786-O cell lines, out of 3032 genes that were upregulated, only 1580 (~50%) were m6A hypermethylated upon SETD2 inactivation. Taken together, results from two independent kidney cell line models isogenic for *SETD2* reveal that while SETD2 loss does not result in global m6A reduction, it does cause marked redistribution of m6A patterns that closely align with changes in gene expression, suggesting an indirect relationship between H3K36me3, m6A, and gene expression.

### Coordinated SETD2 dependent m6A patterns and gene expression changes are enriched in known ccRCC pathways

**Figure 2.**
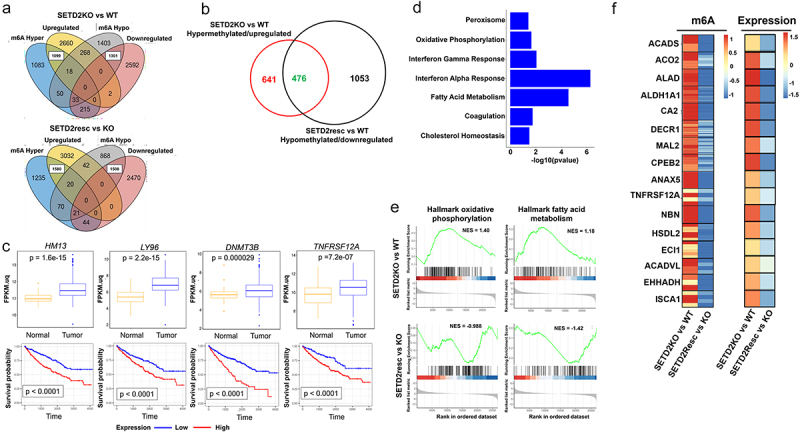
SETD2 dependent m6A and gene expression changes are enriched in known ccRCC pathways. (a) venn diagram depicting overlapping m6A hypermethylated/upregulated (white boxed), m6A hypomethylated/downregulated (white boxed) genes in *SETD2* KO vs WT and SETD2 resc vs KO comparisons. (b) venn diagram depicting how the coordinately regulated SETD2-m6A gene set was identified (overlap region – green, m6A hypermethylated/upregulated expression in *SETD2* KO vs WT, red and m6A hypomethylated/downregulated in SETD2 resc vs *SETD2* KO, black). (c) normalized RNA-seq read counts of representative SETD2-m6A coordinately regulated genes from TCGA – KIRC ccRCC (n = 533) and normal kidney samples (n = 72) (*p*-value = one-way ANOVA, top panel), survival probability with high (*HM13*, *LY96*, *DNMT3B* and *TNFRSF12A*, n = 302, 303, 133 and 403 respectively, red) and low (*HM13*, *LY96*, *DNMT3B* and *TNFRSF12A*, n = 304, 303, 473 and 203 respectively, blue) expression of candidate genes in TCGA’s KIRC dataset (p-value = log – rank test, bottom panel). (d) barplot depicting significantly enriched (*p* value ≤ 0.05) mSigDB hallmark pathways in the coordinately regulated SETD2-m6A gene set. (e) GSEA plots depicting enrichment of hallmark oxidative phosphorylation and fatty acid metabolism pathways in the 786-O *SETD2*KO vs WT and SETD2resc vs KO analysis, NES scores (boxed), *p* value ≤ 0.05, fisher exact test. (f) heatmap depicting log2FoldChange for m6A (left) and expression (right) for select mSigDB hallmark oxidative phosphorylation and fatty acid metabolism genes in SETD2KO vs WT and SETD2resc vs KO comparisons. Scale bar indicates Log2FoldChange.

Considering the robust positive correlation we observed between m6A levels and RNA expression, as well as m6A redistribution closely aligning with gene expression changes overall, we sought to derive a subset of genes where SETD2 status determines both m6A localization and gene expression state. To accomplish this, we identified genes that were upregulated/m6A hypermethylated or downregulated/m6A hypomethylated based strictly on SETD2 loss/rescue dependence. This analysis for 786-O cells revealed 1099 and 1580 genes that were upregulated/m6A hypermethylated with *SETD2* inactivation and rescue, respectively, and 1301 and 1508 genes that were downregulated/m6A hypomethylated with SETD2 inactivation and rescue, respectively (Figure 2a, white boxed regions of the Venn diagrams). Similarly, for RPTEC we found 983 and 624 genes that were coordinately upregulated/m6A hypermethylated with SETD2 inactivation and rescue, respectively, and 747 and 745 genes that were downregulated/m6A hypomethylated with SETD2 loss and rescue, respectively (Supplemental Figure S3A, white boxed regions). We then overlapped genes that were upregulated/m6A hypermethylated with *SETD2* KO, and downregulated/m6A hypomethylated with SETD2 rescue, pinpointing 476 and 307 genes in the 786-O and RPTEC models, respectively, where *SETD2* status was directly linked to loss/gain of both m6A and decreased/increased gene expression (Figure 2b and Supplemental Figure S3B, overlapping regions on the Venn diagrams). We considered these genes high confidence direct SETD2-m6A co-regulated targets. Indeed, these targets included several loci such as *HM13*, *LY96*, *DNMT3B*, *TNFRSF12A, SLC29A3, SERPINH1* and *CHPF* that were upregulated and m6A hypermethylated in the 786-O isogenic model, which were also significantly upregulated in primary ccRCC and associated with poor RCC-specific survival [43–45] (Figure 2c, Supplemental Figure S3C). Furthermore, performing EnrichR pathway analysis on the SETD2-m6A co-regulated gene set revealed enrichment of hallmark oxidative phosphorylation, fatty acid metabolism, and cholesterol homeostasis pathways (*p* ≤ 0.05) in the 786-O cell line model (Figures 2d–e) and hallmark epithelial mesenchymal transition (EMT) and complement pathways in RPTEC (Supplemental Figure S3D), consistent with our earlier studies showing that SETD2 reversibly regulates EMT in RPTEC [31]. While others have shown that *VHL* loss drives lipid droplet accumulation through HIF1α and HIF2α activation [46] our results show increased expression and m6A hypermethylation of genes contributing to lipid peroxidation such as *HSDL2*, *ECI1*, *EHHADH*, and genes like *DECR1* and *ACADVL* that regulate fatty acid beta-oxidation in the mitochondria directly linked to *SETD2* status [47–50] (Figure 2f). Additionally, we identify m6A hypermethylation and upregulation of genes playing prominent roles in oxidative metabolic processes within cells including *ACO2* and *ISCA1* that form essential components of the TCA cycle and electron transport chain, respectively [51–53] (Figure 2f), consistent with the observation that *SETD2* KO 786-O cells show increased mitochondrial respiration rates [30]. Taken together, our results reveal that SETD2-mediated redistribution of m6A and its associated changes in gene expression target essential RCC genes associated with the known metabolic shift in SETD2 deficient cells away from fatty acid accumulation and toward increased peroxisomal and mitochondrial oxidation [30].

### m6A re-distribution in SETD2 KO cells maps to distinct epigenetic states.

**Figure 3.**
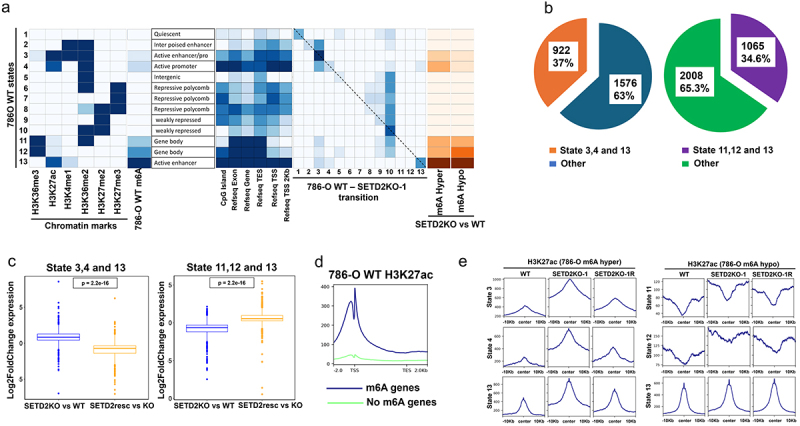
m6A re-distribution in *SETD2* KO cells maps to distinct epigenetic states. (a) ChromHMM modelling of the indicated chromatin marks showing distinct spatial and combinatorial patterns of occupancy (states) in 786-O WT cells with genomic feature enrichment (left). Transition of 786-O WT states to *SETD2*KO-1 (786-O WT- SETD2KO-1 transition, right), *SETD2*KO vs WT m6A hyper and hypomethylated peak enrichment in corresponding 786-O WT states (far right) (b) pie chart showing the fraction of genes in states 3, 4, and 13 corresponding (orange) and not corresponding (blue) to m6A hypermethylated genes, and the fraction of genes in states 11, 12, and 13 corresponding (green) and not corresponding (maroon) to m6A hypomethylated genes. (c) average Log2FoldChange expression for m6A hypermethylated (*SETD2*KO vs WT) genes in states 3, 4, and 13, and m6A hypomethylated (*SETD2*KO vs WT) genes in states 11, 12, and 13 in *SETD2*KO vs WT and SETD2resc vs KO comparisons. (d) tag density plot showing average H3K27ac signal across gene bodies ±2 kb for all genes with at least one detected m6A peak (m6A genes, blue line) and no detected m6A peaks (no m6A genes, green line). (e) tag density plots showing average H3K27ac peak signal centered at m6A hypermethylated (*SETD2*KO vs WT) peaks for states 3, 4, and 13 and m6A hypomethylated (*SETD2*KO vs WT) peaks for states 11, 12, and 13. p-values: one-way ANOVA.

After observing that transcriptome-wide redistribution of m6A upon SETD2 loss was not closely linked with H3K36me3 depletion itself, we explored whether other chromatin-related determinants were more closely linked with m6A redistribution in H3K36me3 depleted cells. To that end, we mapped histone modifications associated with gene bodies (H3K36me2/3), polycomb regulated/repressed regions (H3K27me2/3), and active promoters/enhancers (H3K27ac and H3K4me1) in our 786-O isogenic model. To determine the global architecture of the epigenome in 786-O WT cells and how it relates to m6A, we generated a model which segments the genome in 13 states, based on the combination of histone marks, and observed that m6A was primarily enriched in state 13, which corresponds with the presence of H3K27ac and H3K4me1 (Figure 3a). To investigate how these chromatin states transition with SETD2 loss, we applied the same set of histone modifications in 786-O *SETD2* KO-1 to the WT ChromHMM model to generate the genomic distribution of SETD2 KO histone marks and ultimately, through juxtaposition with the WT distributions, a WT – KO-1 state transition map. The state transition map revealed a transition from WT state 4 to state 3, and states 5–9 and 11–12 to state 10, respectively, indicating a shift in the localization of histone marks in SETD2 KO cells upon global H3K36me3 depletion (Figure 3a). Despite these state transitions signaling significant global chromatin rearrangement with loss of SETD2/H3K36me3, state 13, which had the highest enrichment of m6A in 786-O WT cells, remained consistent even after *SETD2* KO. Overlaying m6A hypermethylated regions onto the 786-O WT 13 state map revealed their enrichment in states 3, 4, 11, 12, and 13, and overlaying m6A hypomethylated regions onto the 786-O WT 13-state map revealed their enrichment in states 11, 12, and 13, with state 13 appearing to represent a hyperdynamic state enriched in both m6A gains and losses (Figure 3a). We also discovered that 37% of all SETD2 KO inactivation m6A hypermethylated genes correspond to states 3, 4, and 13, while 63% of all m6A hypomethylated genes correspond to states 11, 12, and 13 (Figure 3b). Interestingly, SETD2 loss-driven m6A hypermethylation in states 3, 4, and 13 was reversed upon *SETD2* KO rescue, confirming the role of SETD2 in mediating the m6A redistribution observed in these states (Supplemental Figure S4A). Furthermore, we found that nearly 80% of all SETD2-m6A coregulated genes sequestered into states 3, 4, and 13, revealing these states to be hotspots for SETD2 mediated m6A redistribution (Supplemental Figure S4B). While we observe regions with reduced m6A in states 11, 12, and 13 upon *SETD2* KO, these loci did not reverse upon rescue of SETD2 activity, suggesting additional factors determine m6A targeting of this SETD2 regulated gene set or that they represent indirect targets of SETD2 activity (Supplemental Figure S4B). Consistent with our previous findings, we observed an increased gene expression signature for m6A hypermethylated genes corresponding to states 3, 4, and 13, which was reversed upon *SETD2* KO rescue. We observed decreased gene expression in m6A hypomethylated loci corresponding to states 11, 12, and 13 in *SETD2* KO cells that was also reversed with *SETD2* KO rescue (Figure 3c). These findings collectively suggest that SETD2-mediated m6A hyper- and hypomethylation events are sequestered in regions enriched in transcriptional changes.

Having identified high-level enrichment of m6A in state 13 in 786-O WT cells, which was also highly enriched for H3K27ac (Figure 3a), we further examined the interplay between m6A and the active promoter/enhancer mark H3K27ac. We segregated all genes into those with at least one m6A peak and genes with no detectable m6A and scored them for H3K27ac enrichment in the gene body ±2kb. Loci with at least one m6A peak showed more H3K27ac peak signal in both the promoter and gene body than genes with no m6A peaks (Figure 3d). Linear correlation analysis between 786-O *SETD2* KO and 786-O WT differential H3K27ac and differential m6A revealed a robust correlation (*R* = 0.56, *p* < 0.001, Supplemental Figure S4C). To further assess the m6A – H3K27ac association we obtained all m6A hypermethylated peaks in states 3, 4, and 13 and all m6A hypomethylated peaks in states 11, 12, and 13 for aggregated H3K27ac signal. This analysis showed increased H3K27ac levels in *SETD2* KO compared to 786-O WT cells in states 3, 4, and 13, and a subsequent decrease or reversal of these changes upon *SETD2* KO rescue (Figure 3e). Interestingly, the increase in H3K27ac was not observed in *SETD2* KO m6A hypomethylated peaks in states 11, 12, and 13, suggesting m6A is targeted to genomic regions that gain H3K27ac upon SETD2 loss (Figure 3e). Considering that H3K27ac is a key mark that distinguishes active from poised enhancers [54], and in lieu of recent literature suggesting m6A promotes gene activation through transcriptional condensate formation [20], we next examined m6A redistribution to active enhancer regions in *SETD2* KO cells.

### m6A hypermethylation upon SETD2 loss is associated with gain of active enhancer marks H3K27ac and H3K4me1

**Figure 4.**
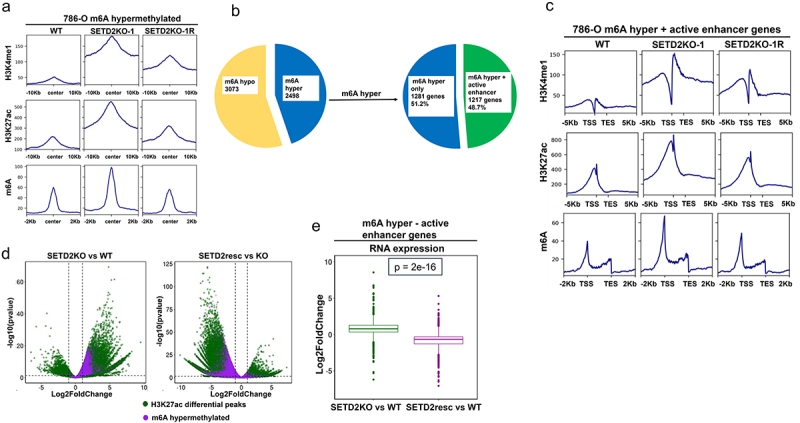
M6A hypermethylation events resulting from SETD2 loss are associated with the gained active enhancer mark H3K27ac. (a) tag density plots showing average H3K4me1, H3K27ac, and m6A signal in 786-O WT, *SETD2*KO-1, and *SETD2*KO-1R centered at m6A hypermethylated peaks. (b) pie chart showing the fraction of m6A hypermethylated genes with gained active enhancer peaks (H3K4me1 and H3K27ac, green) and not showing gained active enhancer peaks (blue). (c) tag density plot showing average H3K4me1, H3K27ac, and m6A peak signal across gene bodies ±5 kb for m6A hypermethylated genes with gained active enhancers. (d) volcano plots depicting differential H3K27ac peaks in the *SETD2*KO vs WT comparison and the SETD2resc vs KO comparison that overlap (purple) and do not overlap (dark green) with m6A hypermethylated peaks. (e) boxplot depicting average Log2FoldChange expression for m6A hypermethylated genes with gained active enhancers for the *SETD2*KO vs WT comparison (dark green) and the SETD2resc vs KO comparison (purple). *p*-values: one-way ANOVA.

To assess whether m6A hypermethylation due to *SETD2* inactivation is enriched in regions characteristic of active enhancers marked by H3K27ac and H3K4me1 chromatin marks, we scored all m6A hypermethylated peaks for H3K27ac and H3K4me1 and found increases in both marks associated with m6A hypermethylated regions (Figure 4a). Furthermore, 48.7% of all m6A hypermethylated genes and 87% of the SETD2-m6A co-regulated gene set described in Figure 2a (for the 786-O model) gained active enhancer peaks upon loss of SETD2 (Figure 4b, Supplemental Figure S5A). This is further supported by increased H3K4me1 and H3K27ac in all m6A hypermethylated and SETD2-m6A coordinately regulated genes in *SETD2* KO compared to WT 786-O cells (Figure 4c, Supplemental Figure S5B) and complete peak-level overlap of m6A hypermethylation events and H3K27ac peaks in *SETD2* KO vs WT 786-O cells (Figure 4d). The increase in H3K27ac and H3K4me1, as well as increased overlap of m6A hypermethylated peaks with H3K27 hyperacetylated peaks, was reversed by rescue of SETD2 function in *SETD2* KO cells, suggesting gained active enhancer marks H3K27ac and H3K4me1 and the associated m6A hypermethylation events are directly mediated by SETD2 status (Figures. 4c,d, Supplemental Figures S5B). In contrast, while we observed an increase in H3K4me1 levels at m6A hypomethylated peaks, there was no increase in H3K27ac associated with these regions, suggesting that active, not poised enhancers are targets for m6A hypermethylation in a SETD2-dependent manner (Supplemental Figure S5C). To investigate the relative enrichment of m6A hypermethylated active enhancer regions in various gene body, cis-regulatory and intergenic loci with SETD2 depletion, we performed GAT analysis. We discovered enrichment of m6A hypermethylated active enhancers in promoter like (Prom) and proximal enhancer like (EnhP), rather than H3K4me3 positive cis-regulatory regions in 786-O SETD2KO–1 cells (Supplemental Figure S5D). Furthermore, we observed the specific enrichment of m6A hypermethylated active enhancers in various gene body regions including 5’-UTR, 3’-UTR, and coding exons, and its de-enrichment in intergenic regions suggesting redistribution of m6A to predominantly gene body enhancers and genic loci in SETD2 depleted 786-O SETD2KO–1 cells (Supplemental Figure S5D). As expected, there was an overall increase in expression of genes in the m6A hypermethylated SETD2-m6A co-regulated gene set with gained active enhancer marks (Figure 4e, Supplemental Figure S5E). Furthermore, m6A hypermethylated and upregulated genes, consistent with a metabolic shift toward fatty acid oxidation and oxidative phosphorylation upon SETD2 inactivation, show H3K27 hyperacetylation, which was reversed, along with m6A methylation and gene expression levels, upon rescue of SETD2 loss (Supplemental Figure S5F).

### Targeting METTL3 in SETD2 deficient cells leads to downregulation of known ccRCC pathways and increased cell death

**Figure 5.**
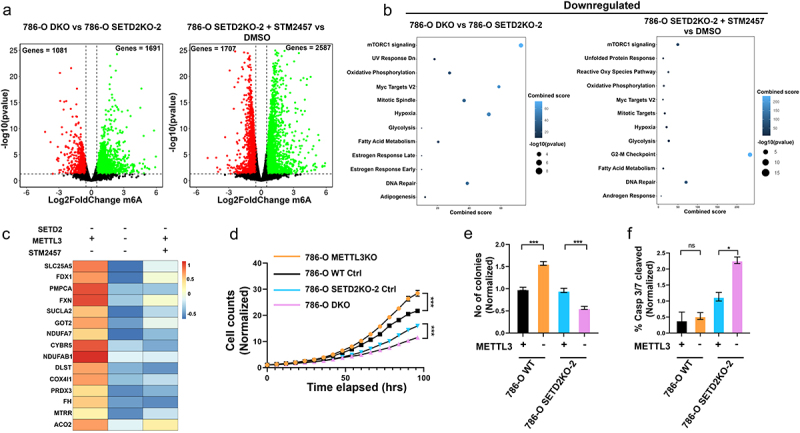
Targeting m6A in *SETD2* KO cells leads to downregulation of known ccRCC pathways and reduced cell viability. (a) volcano plots showing differentially upregulated (Log2FoldChange ≥0.5, *p*-value ≤0.5, green), downregulated (Log2FoldChange ≤0.5, p-value ≤0.05, red), and unchanged (black) genes for 786-O DKO (*SETD2*+*METTL3*KO) and 786-O *SETD2*KO-2 cells treated with 20 µM STM2457 compared to *SETD2*KO-2 control transduced (ctrl) and DMSO vehicle treated cells, respectively. b) EnrichR pathway analysis for downregulated genes in 786-O DKO vs *SETD2*KO-2 ctrl and 786-O *SETD2*KO-2 STM2457 vs DMSO treatments (lighter blue depicts increased enrichment, dot size represents -log10(p-value)). (c) heatmap depicting Log2FoldChange of genes in the 786-O *SETD2*KO-2 (ctrl/dmso) vs WT (ctrl/dmso) comparison, and 786-O *SETD2*KO-2 (DKO/STM2457) vs the 786-O *SETD2*KO-2 control (ctrl/dmso) comparison for genes in the hallmark oxidative phosphorylation pathway. d) cell proliferation curves depicting cell counts for 786-O WT and 786-O *SETD2*KO-2 normalized to 0 hrs (*n* = 6). e) bar graphs depicting number of colonies (*n* = 5) and (f) percent caspase 3/7 cleaved positive cells normalized to sgCas9ctr transduced controls for 786-O WT and *SETD2*KO-2, 786-O WT *METTL3*KO = yellow or ctrl = black, and 786-O DKO = cyan or ctrl = pink, (*n* = 3) *** = *p*-value <0.0001, * = *p*-value ≤0.05, one-way ANOVA.

After observing the enrichment of m6A hypermethylation and upregulation of genes consistent with known metabolic shifts in ccRCC, and given the functional importance of m6A in mRNA metabolism, we assessed the impact of depleting m6A writer METTL3 on gene expression patterns in SETD2 deficient cells. To this end, we inactivated *METTL3* in *SETD2* KO cells using a CRISPR-CAS9 mediated approach or pharmacologic inhibition with the well characterized STM2457 METTL3 small molecule inhibitor that is in clinical trials [55–57]. We generated *METTL3* KOs in both *SETD2* WT and *SETD2* KO 786-O cells (See Methods); these KO clones demonstrated loss of METTL3 protein by WB and reduced global m6A levels in total RNA by ELISA (Supplemental Figures S6A,B). After performing dose optimization for each cell line, we treated 786-O cells with 20 µM METTL3 inhibitor STM2457 for 3 days, which caused global depletion of m6A in total RNA, indicating that the drug is inhibiting its target (Supplemental Figure S6C). To assess the impact of METTL3 genetic inactivation or pharmacologic inhibition on gene expression with or without SETD2 depletion, we performed RNA-seq on the drug treated and *METTL3* KO/control transduced (sgCAS9Ctr, cells transduced with a non-targeting guide RNA) 786-O WT and 786-O *SETD2KO–2* cell lines (Figure 5a). Knockout of *METTL3* resulted in upregulation of 1691 genes and downregulation of 1081 genes; STM2457 treatment resulted in more genes being impacted but with the same modest bias toward upregulation (Figure 5a). Interestingly, EnrichR ontology analysis performed on downregulated genes in 786-O *SETD2*/*METTL3* DKO (786-O DKO) cells revealed enrichment of hallmark oxidative phosphorylation and fatty acid metabolism pathways compared to 786-O *SETD2* KO control transduced cells (786-O SETD2KO–2 Ctrl). This downregulation in oxidative phosphorylation and fatty acid metabolism pathways was only observed in 786-O DKO clones but not the 786-O single *METTL3*KO cells (786-O METTL3KO), suggesting selective targeting of SETD2KO mediated upregulated pathways with METTL3 depletion (Figure 5b, Supplemental Figure S6D). Similarly, downregulated genes in 786-O SETD2KO–2 cells treated with METTL3 inhibitor showed enrichment of hallmark oxidative phosphorylation and fatty acid metabolism pathways compared to DMSO treated cells (Figure 5b). Several TCA cycle promoting genes such as *SUCLA2*, *FH*, *ACO2*, and *DLST* that were upregulated upon *SETD2* KO, became downregulated after METTL3 depletion in *SETD2* KO cells (Figure 5c). Additionally, genes involved in mitochondrial function and ATP production such as *SLC25A5*, *COX4I1, and NDUFA7* that are upregulated with SETD2 inactivation, become downregulated with METTL3 depletion in the *SETD2* KO context (Figure 5c). This finding suggests that the metabolic shift toward oxidative phosphorylation observed with SETD2 inactivation in 786-O cells can be attributed, at least in part, to m6A hypermethylation and that this change is targeted by genetic deletion or pharmacologic inhibition of METTL3 in the SETD2 deficient context. To assess the contribution of ccRCC pathways hypermethylated for m6A in a SETD2 dependent manner to cell growth and viability, we performed cell proliferation and colony formation assays and measured activation of caspase 3/7 with METTL3 depletion in *SETD2* WT and KO 786-O cells. We observed a significant decrease in proliferation rate and colony formation potential in 786-O *SETD2* KO cells with both CRISPR/CAS mediated *METTL3* inactivation and with STM2457 mediated METTL3 inhibition, but not in the 786-O METTL3KO cell line or the STM2457 treated parental 786-O cells (Figures 5d–e, Supplemental Figures S6E, S7A-C). Additionally, METTL3 inactivation or pharmacologic inhibition in *SETD2* KO, but not WT cells resulted in increased caspase 3/7 activation indicative of increased cell death due to apoptosis (Figure 5f, Supplemental Figures S6F, S7D-E). Selective growth detrimental effects of METTL3 inhibition in SETD2 depleted cells compared to SETD2 WT cells was further demonstrated by reduced IC50 concentrations for STM2457 treated 786-O SETD2KO clones compared to 786-O WT cells (Supplemental Figure S7F). Furthermore, in contrast to the *SETD2* KO cells, parental cells displayed modestly elevated growth rate and colony forming potential with *METTL3* inactivation or pharmacologic inhibition (Figures 5d–e) suggesting SETD2 depletion mediated differential m6A patterning, and its associated transcriptional changes, sensitize RCC cells to *METTL3* depletion.

## Discussion

A study by Huang *et al*. indicated an essential role for H3K36me3 in targeting RNA m6A via the METTL14 subunit of the METTL3 – METTL14 m6A methyltransferase complex binding H3K36me3, followed by interaction with RNA polymerase II to deposit m6A on nascent RNA in a co-transcriptional manner using human HepG2 and mouse embryonic stem cells as models [22]. While we did not observe global loss of m6A targeting due to H3K36me3 depletion in isogenic SETD2KO kidney cell line models, our study revealed that loss of SETD2 and the H3K36me3 chromatin mark results in widespread redistribution of m6A throughout the transcriptome, which is dynamic based on SETD2 rescue experiments. This redistribution of m6A closely aligns with changes observed in mRNA transcription, suggesting hotspots that are enriched for both differential mRNA transcription and differential m6A localization that accompany *SETD2* inactivation. Interestingly these hotspots, which we termed SETD2-m6A co-regulated targets, included genes such as *LY96* that not only show higher expression in ccRCC and reduced RCC-specific survival, but have also been implicated in M2 macrophage infiltration in RCC to promote chemotherapy resistance [58]. Other co-regulated genes such as *DNMT3B*, *SERPINH1*, and *CHPF* similarly have been shown to have elevated expression in RCC compared to normal kidney, with CHPF promoting cell proliferation and invasion in ccRCC [59–61]. This suggests SETD2 mediated redistribution of m6A and the associated changes in transcription patterns in key genes contribute to ccRCC progression. Furthermore, we were able to identify regions of the genome linked to other histone modifications enriched for differential m6A levels in a SETD2 dependent manner using our isogenic KO and rescue models to pinpoint the most direct effects of SETD2 activity on these processes. Genomic regions linked to differential m6A hypermethylation due to H3K36me3 depletion were enriched for active enhancer chromatin mark H3K27ac. Further analysis showed that m6A hypermethylation was redistributed to active (H3K4me1 + h3K27ac) rather than poised enhancer (H3K4me1 only) regions due to loss of H3K36me3, suggesting m6A hypermethylated regions are hotspots for active transcription. The redistribution of m6A to gained active enhancer regions we observe suggests that the epigenetic reprogramming resulting from H3K36me3 depletion reshapes m6A methylation patterns throughout the transcriptome.

Our finding demonstrating m6A redistribution on active enhancer regions is reminiscent of recent studies showing enhancer RNA (eRNA) m6A recruiting either BRD4 to promote transcriptional condensate formation or recruiting factors that transform local epigenetic states and promote chromatin accessibility and increased transcription [20,62]. The m6A-enhancer link was further supported by enrichment of m6A hypermethylated active enhancer peaks in annotated cis-regulatory elements corresponding to promoter like, proximal enhancer like, and genic loci within 5’-UTR, 3’-UTR, and coding exons with SETD2 depletion. This comes as an interesting observation since recruitment of the m6A methyltransferase complex to promoters is essential for releasing RNA-pol II into the gene body to facilitate synthesis of nascent RNA transcripts [63]. Moreover, we observed a robust positive correlation between m6A levels and gene expression changes with SETD2 depletion. Taken together, our observations suggest a supportive role of m6A in promoting transcription in an H3K36me3 depleted context, which could be leveraged to repress transcriptional programs characteristic of SETD2 deficient ccRCC. Interestingly, recent studies revealed aberrant activation of enhancers and rampant epigenetic remodeling promoting metastatic transcriptional programs in *SETD2* deficient ccRCC models [9]. We recapitulate similar observations, albeit with increased enhancer m6A levels in genes promoting metabolic reprogramming in *SETD2* deficient ccRCC [30]. The exact mechanism through which m6A levels in active enhancer regions contribute to gene transcription in SETD2 deficient ccRCC remains unknown but is fertile ground for future study.

An emerging hallmark of cancer progression is the reprogramming of bioenergetic pathways to support cell growth and metastasis. CcRCC is characterized by increased expression of genes that promote fatty acid synthesis and glycolysis due to HIF1α and HIF2α activation resulting from the near universal loss of the *VHL* tumor suppressor gene [64]. A recent study that performed metabolic profiling of 138 primary ccRCCs showed enhanced fatty acid biosynthesis and reduced oxidative phosphorylation during the early stages of tumorigenesis, which was reversed during disease progression suggesting that a shift in metabolism is linked to and/or necessary for ccRCC progression, consistent with SETD2 loss being associated with ccRCC progression and reduced relapse free survival [6,9,13,14,65]. In this study, we identified a core set of 476 SETD2-m6A coordinately regulated genes that changed both their expression and RNA methylation status based on loss or rescue of global H3K36me3 levels. Interestingly, a closer examination of these genes revealed their enrichment for processes involved in fatty acid oxidation, TCA cycle, and mitochondrial respiration, suggesting that SETD2 loss-of-function alters pathways regulating energy metabolism, resulting in a shift toward increased oxidative phosphorylation and fatty acid β-oxidation. This is consistent with findings from *Liu et al*. [30], who reported that inactivation of *SETD2* in 786-O cells caused a metabolic shift toward enhanced oxidative phosphorylation, increased fatty acid beta oxidation, and increased TCA cycle activity. Furthermore, the gene expression patterns signaling metabolic reprogramming in *SETD2* deficient ccRCC from lipid accumulation or glycolysis to oxidative phosphorylation or fatty acid beta oxidation are reversed through pharmacologic inhibition or genetic depletion of METTL3 in *SETD2* KO 786-O cells (but not SETD2 proficient cells). This suggests a supportive role for m6A in promoting expression of genes responsible for the metabolic reprogramming observed in SETD2 deficient ccRCC, thereby contributing to disease progression. Taken together, our examination of the functional interplay between SETD2 and METTL3 mediated m6A using both genetic and pharmacologic methods to inhibit METTL3 in *SETD2* WT and mutant contexts revealed that *SETD2* deficient cells were preferentially sensitive to METTL3 loss, potentially through an impact on key metabolic processes like fatty acid oxidation or oxidative phosphorylation that *SETD2* deficient RCC cells have become particularly reliant upon. This, in turn, suggests that targeting m6A in *SETD2* mutant ccRCC could be an efficacious therapeutic strategy.

In this study, we present a unique perspective of *SETD2* deficient ccRCC by not only mapping the transcriptome and epigenome changes resulting from SETD2/H3K36me3 loss, but also integrating effects of SETD2 status on the m6A epitranscriptome. We also present a comprehensive view of how global epigenetic rearrangement influences transcriptomic as well as m6A epitranscriptomic patterns and the influence of m6A RNA modification in regulating gene expression patterns. However, further studies with respect to either transcriptional regulatory or RNA stabilizing effects of m6A mediated by m6A readers will be required to decipher the precise mechanisms through which m6A influences the observed tumor promoting gene expression patterns in *SETD2* mutant ccRCC.

The importance of m6A readers and erasers that mediate RNA processing or metabolism has not escaped our attention. One study showed that expression of the m6A demethylase FTO was elevated in ccRCC compared to normal renal tissue [66,67]. FTO mediated m6A demethylation of glutamine transporter *SLC1A5* and autophagy regulators *ATG5* and *ATG7* stabilizes and destabilizes respectively, their mRNA transcripts through the action of m6A readers, thereby promoting ccRCC by increasing glutamine uptake and inhibiting autophagy [66,67]. The other m6A demethylase, *ALKBH5*, is downregulated in ccRCC, with reduced expression correlating with reduced overall and RCC-specific survival [68]. However, despite observing modestly increased expression of FTO and ALKBH5 in our study with SETD2 depletion in 786-O cells (data not shown), we did not observe global changes in m6A levels with SETD2 depletion or rescue in 786-O cells as is typically observed upon augmented expression of the m6A demethylases [67,69]. M6A readers like the *YTHDF1* − 3 paralogs, have a transcript translation or decay promoting effect on m6A modified nascent transcripts by either relieving ribosomal stalling or recruiting the CCR4-NOT deadenylating complex respectively [24,70]. In contrast, m6A readers of the *IGF2BP*1–3 family primarily elicit stabilizing effects on mRNA transcripts by binding the m6A modification via their KH domain and forming stable RNA-protein complexes that prevent mRNA decay in the cytoplasm [19,21,71]. These functions of m6A mediated by cytosolic readers largely effect transcript half-life in the cytoplasm thereby promoting protein translation or transcript degradation. M6A readers have been implicated in ccRCC initiation/progression. For example, *YTHDF2*-mediated recognition and degradation of *ITGB4* transcripts prevent cell migration and invasion in ccRCC [72]. *IGF2BP* paralogs are highly expressed in RCC compared to normal kidney and act to promote cell migration and proliferation in ccRCC cell lines when ectopically expressed [73]. In addition, *IGF2BP1* and *IGF2BP3* stabilize m6A modified transcripts *LDHA* and *CDK4* to promote aerobic glycolysis and cell proliferation, respectively, in ccRCC cell lines [29,74]. Differential m6A patterning observed upon H3K36me3 depletion may indeed render m6A differentially methylated transcripts susceptible to the action of cytosolic m6A readers that could stabilize or destabilize the mRNA transcript thereby effecting their translational output. Considering the substantial evidence indicating widespread m6A redistribution to varied genomic loci upon *SETD2* depletion, as well as the strong positive relationship between m6A levels and gene expression we observed, we focused our studies on uncovering links between differential m6A patterning and global epigenetic rearrangements as it pertains to the influence of m6A in transcriptional regulation. Future studies examining the role of m6A readers in mediating the expression and phenotypic changes in RCC cells with *SETD2* and/or *METTL3* inactivation are certainly warranted.

## Supplementary Material

Supplemental Material

## Data Availability

MeRIP-seq, RNA-seq, and ChIP-seq data generated in this study are available for download in NCBI GEO accession numbers GSE276357 and GSE276371.
