# Supplementary material for: Deciphering the interplay between SETD2 mediated H3K36me3 and RNA N6-methyladenosine in clear cell renal cell carcinoma (ccRCC)
Source: Epigenetics. 2025 Jan 28;20(1):2456418. doi: 10.1080/15592294.2025.2456418 (PMC11776469; doi:10.1080/15592294.2025.2456418)
Supplement: Supplemental Material [file KEPI_A_2456418_SM0931.docx]

**Supplemental Material for Shaikh *et al*., ‘Deciphering the interplay between SETD2 mediated H3K36me3 and RNA N6-methyladenosine in clear cell renal cell carcinoma (ccRCC)’**

**Supplemental Figure Legends.**

**Supplemental Fig. S1: METTL3 links to ccRCC in TCGA-KIRC and characterization of H3K36me3 and m6A localization patterns in ccRCC isogenic models.** A-B) Normalized RNA-seq read counts for *METTL3* expression from TCGA – KIRC ccRCC (n = 533) and normal (n = 72) samples (p-value = one-way ANOVA) and survival probability with high (n = 267, red) and low (n = 268, blue) expression of *METTL3* in TCGA’s KIRC dataset, (p-value = log rank test). C) Heatmap depicting H3K36me3 peaks across all genes in 786-O and RPTEC isogenic cell lines (WT, *SETD2*KO-1 and *SETD2*KO-2, and SETD2KO-1R). D) Tag density plot showing average H3K36me3 and m6A peak signal across gene bodies + 5 kb for RPTEC WT, *SETD2*KO-1 and *SETD2*KO-2, and SETD2KO-1R. E) Representative western blot of the indicated histone modifications in 786-O isogenic cell lines. F) Consensus m6A motif RRACH with methylated adenosine highlighted in red enriched in the top 3000 786-O WT m6A peaks derived from MeRIP-seq. G) Total number of m6A peaks called for RPTEC WT, *SETD2*KO-1, *SETD2*KO-2 and SETD2KO-1R mRNA (n = 2). RT-qPCR quantification showing fold enrichment (2^(-ΔΔct)^) of *METTL3* expression in H) 786-O and RPTEC *SETD2*KO-1 and *SETD2*KO-2 isogenic lines compared to *SETD2* WT cells (n = 3), and I) *SLC29A3*, *SERPINH1*, *HM13* and *CHPF* expression in 786-O *SETD2*KO-1 and SETD2KO-1R compared to WT cells (n = 3).

**Supplemental Fig. S2: m6A and gene expression are positively correlated**. A) Volcano plots depicting differential m6A hypermethylated peaks (Log2FoldChange >= 0.5, p-value <= 0.05, orange), m6A hypomethylated peaks (Log2FoldChange <= 0.5, p-value <= 0.05, blue) and unchanged (black, top panel) and differential gene expression, upregulated (Log2FoldChange >= 0.5, p-value <= 0.05, green), downregulated (Log2FoldChange <= 0.5, p-value <= 0.05, red), and unchanged, black, bottom panel) in RPTEC *SETD2*KO vs WT and SETD2resc vs KO comparisons. Gene level view of *POU2F2* and *MAL* showing m6A and H3K36me3 levels in RPTEC WT (blue), *SETD2*KO-1 (red), and SETD2KO-1R (green), with tan colored boxes highlighting called m6A peaks. C) Correlation plot comparing Log2FoldChange m6A and Log2FoldChange expression for *SETD2*KO vs WT (786-O, R = 0.47, p < 0.001 and RPTEC, R = 0.58, p < 0.001) and SETD2resc vs KO (786-O, R = 0.71, p < 0.001 and RPTEC, R = 0.48, p < 0.001) comparisons in 786-O and RPTEC cell lines.

**Supplemental Fig. S3: The SETD2-m6A co-regulated gene set in RPTEC lines is enriched for hallmark epithelial mesenchymal transition (EMT).** A) Venn diagram depicting overlapping m6A hypermethylated/upregulated (white boxed) and m6A hypomethylated/downregulated (white boxed) genes in *SETD2*KO vs WT and SETD2resc vs KO comparisons in RPTEC. B) Venn diagram depicting the coordinately regulated SETD2-m6A gene set (green) (m6A hypermethylated/upregulated in *SETD2*KO vs WT, red and m6A hypomethylated/downregulated in SETD2resc vs KO, black) for RPTEC. C) Normalized RNA-seq read counts of representative SETD2-m6A coordinately regulated genes from TCGA – KIRC tumor (n = 533) and normal samples (n = 72) (p-value = one-way ANOVA, top panel), survival probability with high (*SERPINH1*, *SLC29A3* and *CHPF*, n = 294, 295 and 294, respectively) (red) and low (*SERPINH1*, *SLC29A3* and *CHPF*, n =293, 292 and 293, respectively) (blue) expression of candidate genes in TCGA’s KIRC dataset (p-value = log – rank test, bottom panel). D) Enrichr pathway analysis performed on the coordinately regulated SETD2-m6A gene set for the RPTEC model (lighter blue depicts increased combined score (enrichment), dot size depicts -log10(pvalue)).

**Supplemental Fig. S4: m6A RNA methylation and H3K27 acetylation are positively correlated across *SETD2* KO vs WT comparisons.** A) Tag density plots showing average m6A signal in 786-O WT, *SETD2*KO-1, and SETD2KO-1R centered at m6A hyper (*SETD2*KO vs WT) and hypomethylated (*SETD2*KO vs WT) peaks for corresponding states. B) Barplot depicting the percent contribution of states 3, 4, 11, 12, and 13 to the coordinately regulated SETD2-m6A gene set. C) Correlation plot comparing Log2FoldChange H3K27ac and Log2FoldChange m6A for the *SETD2*KO vs WT comparison (R = 0.56, p < 0.001).

**Supplemental Fig. S5: SETD2-m6A co-regulated genes show enrichment of active enhancer chromatin marks H3K27ac and H3K4me1.** A) Pie chart showing the fraction of the coordinately regulated SETD2-m6A gene set with (yellow) and without (purple) gained active enhancers. B) Tag density plot showing average H3K4me1, H3K27ac, and m6A peak signal across the gene body + 5 kb for the coordinately regulated SETD2-m6A gene set. C) Tag density plot showing average H3K27ac, H3K4me1 and m6A peak signal across gene bodies + 5 kb for m6A hypomethylated genes. D) GAT analysis demonstrating the relative Log2FoldChange enrichment of m6A hypermethylated active enhancer regions to various gene body (5’-UTR, 3’-UTR, coding exon, intron), cis-regulatory regions (EnhP, +/- 2kb of the TSS, EnhD, +2kb through the gene body) and Promoter-like, and intergenic regions. E) Boxplot depicting average expression Log2FoldChange for coordinately regulated SETD2-m6A genes with gained active enhancer mark for the *SETD2*KO vs WT (red) comparison and the SETD2resc vs KO (black) comparison, p-values: one-way ANOVA. F) Heatmap depicting H3K27ac, m6A, and expression Log2FoldChange for coordinately regulated SETD2-m6A genes for *SETD2*KO vs WT and SETD2resc vs KO comparisons in 786-O. Scale bar indicates log2FoldChange.

**Supplemental Fig. S6: Pharmacologic inhibition or CRISPR/CAS9 mediated genetic inactivation of *METTL3* leads to reduced m6A levels and a selective reduction in cell viability and colony formation in *METTL3*/*SETD2* DKO cells.** A) Western blot analysis of 786-O WT Ctrl and *METTL3*KO (clones KO1, KO2, and KO3), *SETD2*KO-2 Ctrl, and DKO (clones DKO1 and DKO2) cells showing METTL3 protein levels. M6A ELISA showing global m6A levels in total RNA from B) 786-O *METTL3*KO and DKO cell lines relative to their respective control lines (n = 2), and C) 786-O *SETD2*KO-2 cells treated with 20µM STM2457 or DMSO vehicle control (n = 2). D) Enrichr pathway analysis performed on 786-O *METTL3*KO vs WT-Ctrl downregulated genes (lighter blue depicts increased combined score (enrichment), dot size depicts -log10(pvalue)). significance values represent respective comparisons. *** = p-value < 0.0001, ** = p-value <= 0.001, * = p-value <= 0.05, one-way ANOVA. E) Representative pictures depicting clonogenic assays from 786-O *METTL3*KO and WT-Ctrl as well as 786-O DKO and *SETD2*KO-2 Ctrl cells. F) Representative flow cytometry scatterplots depicting percent caspase 3/7 positive cells in 786-O WT, *METTL3*KO, and Ctrl, as well as 786-O DKO and *SETD2*KO-2 Ctrl cells.

**Supplemental Fig. S7: Pharmacologic inhibition of METTL3 in 786-O *SETD2* KO cells leads to a selective decline in cell viability and colony forming potential.** A) Cell proliferation curves depicting averaged cell counts for 20µM STM2457 and DMSO treated 786-O *SETD2*KO-2 cells normalized to 0 hrs (n = 6). B) Bar graphs depicting averaged number of 786-O *SETD2*KO-2 colonies treated with 20µM STM2457 (red) normalized to DMSO vehicle treated controls (blue) cells (n = 5) and C) representative images of clonogenic assays. D) Percent caspase 3/7 positive 786-O *SETD2*KO-2 cells after treatment with 20µM STM2457 (red) normalized to DMSO vehicle treated controls (blue) (n = 2). E) Representative flow cytometry scatterplots depicting percent caspase 3/7 positive cells for the indicated cells/treatments. F) IC50 curves for 786-O WT (blue) and *SETD2*KO-1 (red), and *SETD2*KO-2 (green) cells treated with 20µM STM2457 (n = 3). X-axis = log10 concentrations, Y-axis = Absorbance (490nm). Significance values represent respective comparisons. **** = p-value < 0.0001, *** = p-value <= 0.001, ** = p-value <= 0.01, one-way ANOVA.

**Supplemental Fig. S8: In-house generated CRISPR-CAS9 expressing lentiviral vector with cloned METTL3gRNA.** METTL3 gRNA cloned into the pSICO lentiviral vector expressing METTL3 gRNA/CRISPR-CAS9 under control of the mouse U6 promoter.

**Supplemental table S1 QPCR primers**

| **Gene** | **Forward primer** | **Reverse primer** |
| --- | --- | --- |
| METTL3 | CGTAGCTGAGGTTCGTTCCA | TTGCGAGTGCCAGGAGATAG |
| SERPINH1 | GATCAACTTCCGCGACAAGC | ACATGGCGTTGACTAGCAGG |
| SLC29A3 | ACAGAACCACAAGCAGCAGT | CAGTAGACTGCCAATGCCCA |
| HM13 | CCCTCAGCGATCCGCATAA | AGAAGATGGGCAGCAGCG |
| CHPF | GTTCTTCCTGGTGCCTGACA | CTCTCCGCCGATGAAGTCC |

**Supplemental table S2 MeRIP-QPCR primers**

| **Gene** | **Forward primer** | **Reverse primer** |
| --- | --- | --- |
| SERPINH1 | CCGAGCAATCTGGGGGTG | ATTTGTAGGCTCTGGCTCGG |
| SLC29A3 | GCACAAGTAAGAGCAAGAGGT | GCAGCCCTGTCAACTAAACAA |
| HM13 | GAGCCCAGTCACCAAGGAGA | TTGGACTCTCCCAATCCATGC |
| CHPF | CAGCCTATGACCTGTTGTGGAC | CAGATGAGCTAGCGGAGGCA |

**Supplemental table S3 Cell lines and sequencing experiments**

| **Cell line** | **Experiment** | **n/experiment** |
| --- | --- | --- |
| 786-O WT | RNA-seq/MeRIP-seq/Chip-seq | 2 |
| 786-O SETD2KO-1 | RNA-seq/MeRIP-seq/Chip-seq | 2 |
| 786-O SETD2KO-2 | RNA-seq/MeRIP-seq/Chip-seq | 2 |
| 786-O SETD2KO-1R | RNA-seq/MeRIP-seq/Chip-seq | 2 |
| RPTEC WT | RNA-seq/MeRIP-seq/Chip-seq | 2 |
| RPTEC SETD2KO-1 | RNA-seq/MeRIP-seq/Chip-seq | 2 |
| RPTEC SETD2KO-2 | RNA-seq/MeRIP-seq/Chip-seq | 2 |
| RPTEC SETD2KO-1R | RNA-seq/MeRIP-seq/Chip-seq | 2 |
| 786-O METTL3KO | RNA-seq | 2 |
| 786-O WT Ctrl | RNA-seq | 2 |
| 786-O DKO | RNA-seq | 2 |
| 786-O SETD2KO-2 Ctrl | RNA-seq | 2 |
| 786-O WT STM2457 20µM | RNA-seq | 2 |
| 786-O WT DMSO | RNA-seq | 2 |
| 786-O SETD2KO-2 STM2457 20µM | RNA-seq | 2 |
| 786-O SETD2KO-2 DMSO | RNA-seq | 2 |

**Supplemental table S4 Antibodies and experiments**

| **antibody** | **company** | **clone/cat.#** | **Experiment** |
| --- | --- | --- | --- |
| α-H3K36me3 | Active Motif | 61101 | Western Blot/ChIP-seq |
| α-H3K36me2 | Cell Signaling Tech. | C75H12 | Western Blot/ChIP-seq |
| α-H3K36me1 | abcam | ab9048 | Western Blot |
| α-H3K27me3 | Cell Signaling Tech. | C36B11 | ChIP-seq |
| α-H3K27me2 | abcam | ab26484 | ChIP-seq |
| α-H3K27ac | Cell Signaling Tech. | D5E4 | ChIP-seq |
| α-H3K4me1 | Cell Signaling Tech. | D1A9 | ChIP-seq |
| α-H3total | abcam | ab1791 | Western Blot |
| α-METTL3 | Proteintech | 15073-I-AP | Western blot |
